# Are all bacterial strains required by OECD mutagenicity test guideline TG471 needed?

**DOI:** 10.1016/j.mrgentox.2019.503081

**Published:** 2019-08-09

**Authors:** Richard V. Williams, David M. DeMarini, Leon F. Stankowski, Patricia A. Escobar, Errol Zeiger, Jonathan Howe, Rosalie Elespuru, Kevin P. Cross

**Affiliations:** a Lhasa Limited, 2 Canal Wharf, Leeds, LS11 5PS, United Kingdom; b U.S. Environmental Protection Agency, Research Triangle Park, NC, 27511, United States; c Charles River Laboratories - Skokie, LLC, 8025 Lamon Ave., Skokie, IL, 60077, United States; d Merck & Co., West Point, PA, 19486, United States; e Errol Zeiger Consulting, 800 Indian Springs Road, Chapel Hill, NC, 27514, United States; f Glaxo Smith Kline R&D, Park Road, Ware, Hertfordshire, SG12 0DP, United Kingdom; g U.S. Food and Drug Administration, 10903 New Hampshire Avenue, Silver Spring, MD, 20993, United States; h Leadscope, Inc., 1393 Dublin Rd, Columbus, OH, 43215, United States

**Keywords:** Bacterial mutagenicity, Salmonella, Ames assay, *E. coli* WP2

## Abstract

The International Workshop on Genotoxicity Testing (IWGT) meets every four years to seek consensus on difficult or conflicting approaches to genotoxicity testing based upon experience, available data, and analysis techniques. At the 2017 IWGT meeting in Tokyo, one working group addressed the sensitivity and selectivity of the bacterial strains specified in the Organization for Economic Cooperation and Development (OECD) Test Guideline TG471 to recommend possible modification of the test guideline. Three questions were posed: (1) Although TA100 is derived from TA1535, does TA1535 detect any mutagens that are not detected by TA100? (2) Among the options of *Salmonella* TA1537, TA97 or TA97a, are these strains truly equivalent? (3) Because there is a choice to use one of either *E. coli* WP2 *uvrA*, *E. coli* WP2 *uvrA* pKM101, or *Salmonella* TA102, are these strains truly equivalent? To answer these questions, we analyzed published bacterial mutation data in multiple strains from large (> 10,000 compound) databases from Leadscope and Lhasa Limited and anonymized data for 53 compounds tested in TA1535 and TA100 provided by a pharmaceutical company. Our analysis involved (1) defining criteria for determining selective responses when using different strains; (2) identifying compounds producing selective responses based upon author calls; (3) confirming selective responses by visually examining dose-response data and considering experimental conditions; (4) using statistical methods to quantify the responses; (5) performing limited additional direct-comparison testing; and (6) determining the chemical classes producing selective responses. We found that few mutagens would fail to be detected if the test battery did not include *Salmonella* strains TA1535 (8/1167), TA1537 (2/247), TA102 (4/46), and *E. coli* WP2 *uvrA* (2/21). Of the mutagens detected by the full TG471 strain battery, 93% were detected using only strains TA98 and TA100; consideration of results from *in vitro* genotoxicity assays that detect clastogenicity increased this to 99%.

## 1. Introduction

The bacterial reverse-mutation assay has been used for the evaluation of the mutagenic potential of pharmaceuticals, industrial chemicals, and environmental samples for nearly half a century, using mutant strains of *Salmonella typhimurium* (Ames assay) and *Escherichia coli* (WP2 strains). In this test system, the mutations are measured as reversion to histidine prototrophy of *Salmonella* test strains that are histidine auxotrophs [1], and as reversion to tryptophan prototrophy of an *E. coli* test strain that is a tryptophan auxotroph [2]. It is typically the first mutagenicity assay used in a test battery consisting of assays in bacterial strains, mammalian cells, and rodents to evaluate agents for genotoxicity, both for research purposes and to comply with regulatory requirements [3,4]. Which assays should be used for genotoxicity evaluation, how they should be used, and how they should be interpreted is the subject of continuous discussion [5].

To advance those discussions, the International Workshop on Genotoxicity Testing (IWGT) is held every four years in conjunction with the International Conference on Environmental Mutagens (ICEM) [6]. The IWGT brings together world experts from industry, academia, and government to address topics of current importance for genetic toxicologists, pharmaceutical and chemical industries, and the relevant public health and regulatory agencies. The most recent IWGT, the 7th, was held on 8–10 November 2017 in Tokyo, Japan, where a variety of topics were discussed. This paper describes one of those topics, which was the analyses conducted and conclusions that addressed and provided recommendations for potential revisions to the current Organization for Economic Cooperation and Development (OECD) bacterial mutagenicity Test Guideline TG471 [7], which has not been revised for more than 20 years.

The questions regarding which bacterial strains should be included for routine bacterial reverse-mutation assays, as well as how they should be conducted and interpreted, were addressed at the first IWGT, held in Melbourne, Australia, in 1993 [8]. Most of the recommendations from this meeting were incorporated into the revised version of the OECD test guideline TG471, which was issued in 1997 [7,8]. OECD TG471 recommends that at least five strains of bacteria be used in the following combination: *Salmonella* TA1535; *Salmonella* TA1537 or TA97 or TA97a; *Salmonella* TA98; *Salmonella* TA100; and *Salmonella* TA102 or *E. coli* WP2 *uvrA* or *E. coli* WP2 *uvrA* pKM101.

These strains were selected because initial and emerging molecular analyses indicated that, collectively, they permitted the recovery of all six classes of base-substitution mutations, three classes of frameshift mutations, and some small deletions [1,2,9,10]. Mutations at AT sites were recoverable in the *E. coli* WP2 strains as well as in *Salmonella* TA102, whereas all the other strains permitted recovery of mutations only at GC sites. In *Salmonella*, the addition of the pKM101 plasmid generally enhanced mutagen sensitivity and resulted in the induction by some mutagens of complex mutations (both frameshifts and base substitutions) recoverable in TA98 [10]. Genomic hybridization showed that the different Δ*uvrB* mutations generated to produce the various *Salmonella* strains resulted in the deletion of 47–199 genes [11], with deletion of some genes being more significant than the removal of *uvrB* in altering the mutagenic response of the strain [12]. Although the strains varied by their alleles and DNA repair backgrounds, there was substantial redundancy among some strains to permit the recovery of some of the same classes of mutations.

In the time since the publication of OECD TG471, the database of *Salmonella* mutagenicity assay data has expanded to > 10,000 compounds, and the storage of those data in searchable, electronic databases now permits analyses that were not possible 25 years ago. Thus, it was considered both sensible and timely to review whether the recommended strain battery is consistent with current experience. To this end, we posed three questions. (1) Although TA100 is derived from TA1535 and generally detects more mutagens than TA1535, does TA1535 detect any mutagens that are not detected by TA100? Both strains detect base-pair substitutions at the same GC base pairs; however, TA100 contains pKM101, which confers error-prone DNA repair. (2) Because there is a choice to use one of either *Salmonella* TA1537, TA97 or TA97a for detection of frameshift mutagens, are these strains truly equivalent? (3) Because there is a choice to use one of either *E. coli* WP2 *uvrA*, *E. coli* WP2 *uvrA* pKM101, or *Salmonella* TA102 for detection of base substitutions at AT base pairs, are these strains truly equivalent?

We addressed these questions by first obtaining data from one of three sources: (1) two commercial electronic databases containing mutagenicity data on > 10,000 compounds from the open literature, (2) anonymized, proprietary data from a pharmaceutical company, and (3) generating test data ourselves where literature data were unsuitable for comparison. We then performed a series of pairwise comparisons of various compounds in the strains using the qualitative calls of the authors (who generally did not use statistical analyses and who mostly used a 2- or 3-fold rule) to determine if any agents were mutagenic in only one strain of the pair being compared. Where possible we also evaluated whether a compound that was mutagenic in only one of the related strains was also mutagenic in any other OECD-recommended strains or other commonly used genotoxicity assays.

In addition to using the study calls reported by the authors, which were almost exclusively based on non-statistical analyses, we also addressed the three questions above quantitatively by calculating linear regressions and determining the slopes (mutagenic potencies) over the linear portions of the dose-response curves, using a trend-test (*P* ≤ 0.05) for significance. This analysis was limited to chemicals where we had access to suitable dose-response data. In addition, we also checked if strains preferentially detected compounds of certain chemical classes or structures. The results of these various analyses showed the potential impact of eliminating some strains from OECD TG471 in terms of its ability to detect bacterial mutagens.

## 2. Materials and methods

### 2.1. Data collection

We obtained data from two electronic databases, from an anonymized set of results for strains TA100 and TA1535, and we generated test data for 10 additional compounds ourselves. From electronic databases, we extracted data from the Vitic database 2.6.2 (Lhasa Limited, Leeds, UK; https://www.lhasalimited.org/products/vitic.htm) and the Leadscope 2017 genetic toxicity database (Leadscope, Inc., Columbus, OH; https://www.leadscope.com/). We obtained the data in three batches for compounds tested in (1) both *Salmonella* TA100 and TA1535, (2) at least two of *Salmonella* TA97, TA97a, or TA1537, and (3) at least two of *Salmonella* TA102, *E. coli* WP2 *uvrA*, or *E. coli* WP2 *uvrA* pKM101. We then curated the data extracted from the databases by excluding studies that used assays other than the standard plate-incorporation protocol or pre-incubation protocol, such as heat-inactivated S9, photo-mutagenicity assays, desiccator studies for volatiles, mini-assays, micro-suspension assays, the Prival modification for azo dyes, *etc.*

Additional data that were not in the open literature or in the Leadscope or Lhasa databases were provided by a pharmaceutical company for analysis. These were anonymized, proprietary data for 53 compounds in TA1535 and TA100 that were viewed by the company as mutagenic only in TA1535 and not in any of the other strains used in TG471. The basis for a positive call was the 2-fold rule, where a dose-related increase was observed, and the numbers of revertants (rev) per plate equaled or exceeded a 2-fold increase relative to the background. We analyzed these data statistically (see below), and they comprised an additional dataset from which to assess the proportion of compounds that were putative mutagens in TA1535 but not in TA100.

Finally, five compounds each from the TA1537/TA97 and *E. coli* WP2/TA102 datasets were selected for re-testing because the results were obtained from different laboratories, which could be a confounding factor in the analysis. Using the OECD TG471 procedures, we evaluated those ten compounds ourselves in the plate-incorporation and/or liquid pre-incubation protocols in Charles River Laboratories - Skokie.

### 2.2. Assessing selectivity

**Fig. 1.**
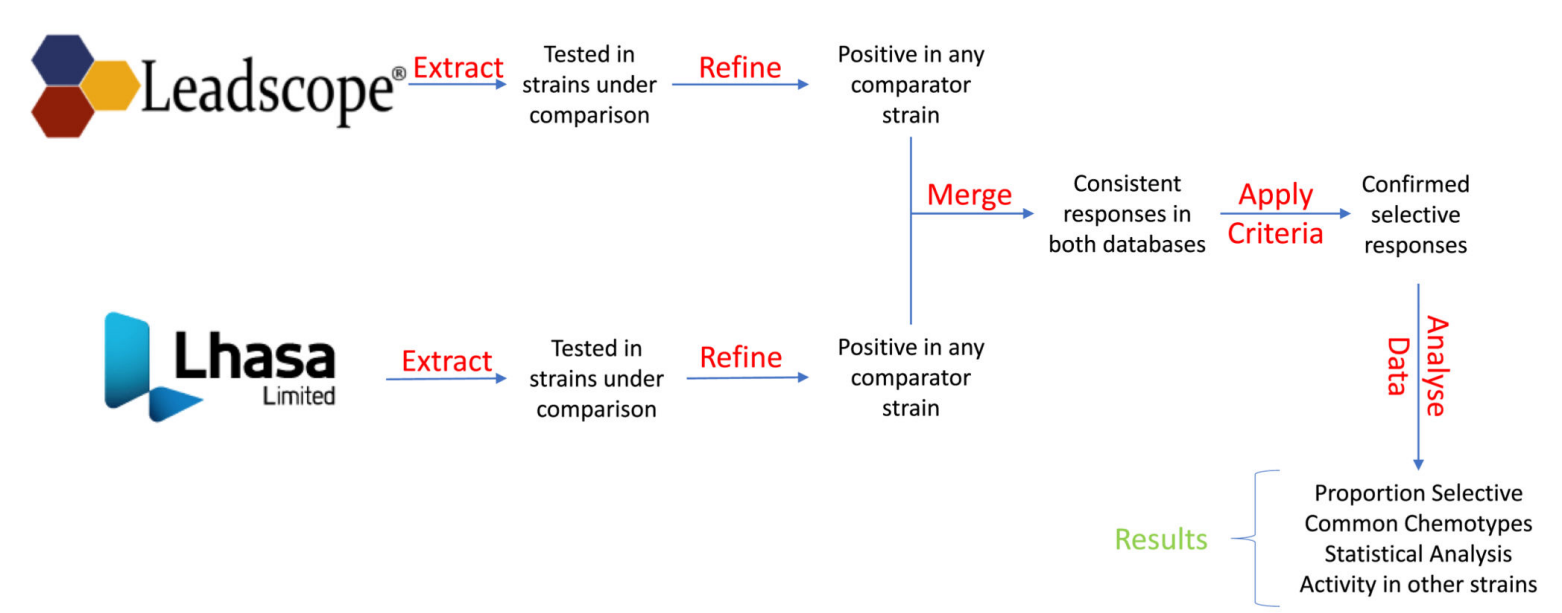
Schematic diagram showing the procedure employed for curating and analyzing the data to determine the percentage of compounds that are mutagenic in only one strain of a series of pairwise comparisons of strains.

A schematic outline of our analysis is shown in Fig. 1 and is described below. We performed a high-level comparison of the data in each dataset to identify compounds that were mutagenic in either both strains or in one strain of the pair being compared based on the authors’ qualitative calls as recorded in the databases. To ensure consistency in this effort, we crafted the following definitions to direct our decisions:

- Selective response: mutagenic in only one of the two strains being compared, but may be mutagenic, non-mutagenic, or untested in other standard Ames strains.
- Unique response: mutagenic in only one of the two strains being compared and not mutagenic in any other standard Ames strains where a test had been run.
- Test strain: the strain that generated the fewest selective responses in the pairwise comparison.
- Comparator strain: the strain that generated the highest number of selective responses in the pairwise comparison.

After identifying the compounds that gave responses that were either selective (mutagenic in only one strain of the pair) or common (same response in both strains) in the Lhasa and Leadscope datasets, we merged the results from the two datasets and performed a comprehensive manual review of all the data to ensure uniform application of our selection criteria. If we found differences in the calls for a compound between the two databases, we treated such studies as we did for conflicting studies within a single database, *i.e.*, we marked such results as not reproducible. Where possible, this involved examining the primary literature to capture the dose-response data and test conditions for all selective responses in the strains. When examining the literature, we considered the following factors to explain reported selective responses, and if such factors could be eliminated, then we included the data in the final dataset:

- Testing protocols, *e.g.*, plate incorporation or pre-incubation *versus* desiccator protocol.
- Metabolic activation conditions, *e.g.*, both the concentration of S9 in the S9 mix, *e.g.*, 5% *versus* 30%, and the species from which the S9 was made.
- Highest doses tested or dose-spacing.
- Response within a single strain, *i.e.*, where there were inconsistent responses within the test or comparator strains.
- No obvious solvent-mediated activation or deactivation.
- No obvious issues with sample purity (although purity issues were rarely reported).

As indicated above, the working group addressed the issue of whether the data for any pairwise comparison were from the same study or laboratory, or whether data for a compound were generated in one strain in one laboratory and in another strain in a different laboratory. For the TA100 *versus* TA1535 (TA100-TA1535) dataset, this was not an issue because most compounds had been tested in both strains in the same study (as prescribed in OECD TG471). However, application of this criterion for the TA97 *versus* TA1537 (TA97-TA1537) and *E. coli* WP2 *versus* TA102 (*E. coli*-TA102) datasets would have reduced the size of those datasets to such an extent as to make comparisons impossible. This prompted the re-tests as described above.

### 2.3. Statistical analysis

Where suitable dose-response data were available, we generated linear regressions and calculated the slopes (mutagenic potencies) expressed as revertants/μg (rev/μg) over the linear portion of the dose-response curves using Prism (GraphPad, San Diego, CA). The analysis also produced r^2^- and *P*-values (based on a trend test); we used *P* < 0.05 to determine significance. We compared mutagenic potencies by using two-tailed t-tests (GraphPad, San Diego, CA), setting *P* < 0.05 for significance.

## 3. Results and discussion

### 3.1. Overall comparisons from the Leadscope and Lhasa databases

**Fig. 2.**
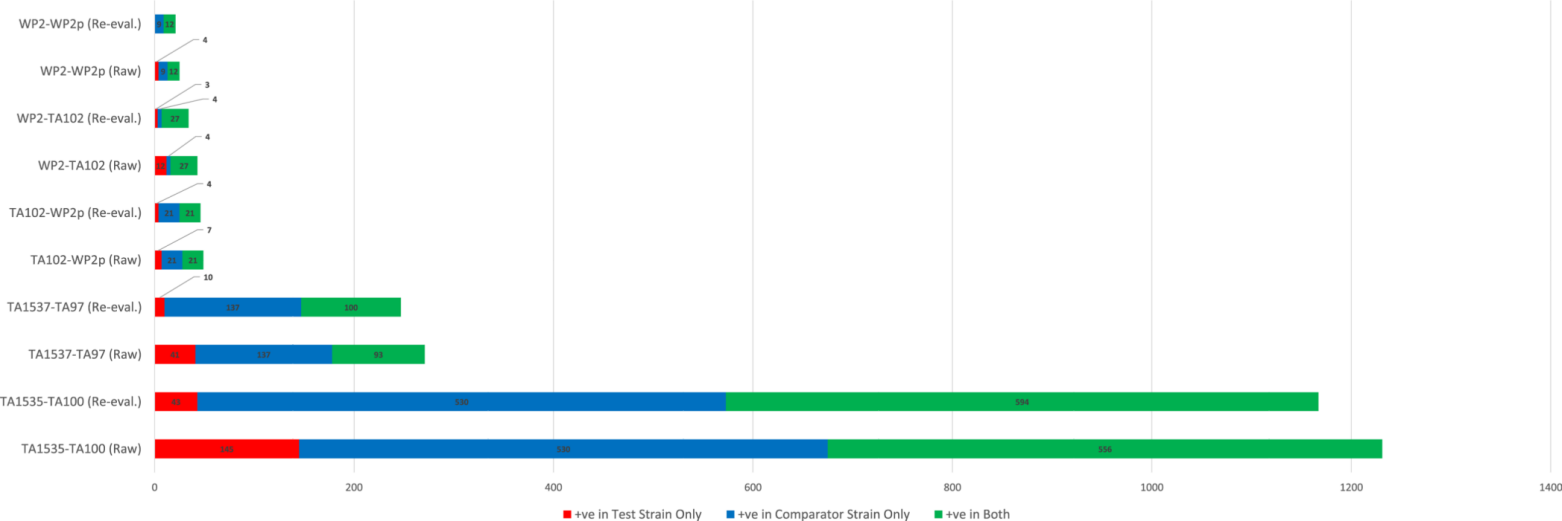
Plot showing the distribution of positive responses in test and comparator strains before and after data were re-evaluated.

Table 1 shows the number of compounds in each pairwise comparison based on the initial query of the Leadscope and Lhasa databases. The results indicated that there were ∼100 to > 1000 compounds in each database for each pairwise strain comparison. After curation of the data from both databases and assessing selectivity as described in the Materials & Methods, we merged the two datasets and applied the selection criteria to produce Table 2. Positive responses in test and/or comparator strains before and after the application of the selection criteria are plotted in Fig. 2. This analysis, based on the authors’ calls (none of which involved statistical analyses), indicated that only ∼4% of compounds were mutagenic in TA1535 and not in TA100, or mutagenic in TA1537 and not in TA97. Only ∼8% of compounds were positive in either TA102 or *E. coli* WP2 *uvrA* and not in *E. coli* WP2 *uvrA* pKM101.

In all cases, selective responses in test strains were observed for < 10% of the compounds, indicating that for the pairwise comparisons, the test strains (TA1535, TA1537, and TA102) showed less sensitivity to mutagens than did the comparator strains (TA100, TA97, and WP2 *uvrA* with or without pKM101), respectively. In most cases, the high frequencies of selective responses (∼50%) occurred in the comparator strains, and only in the case of TA102 *versus* WP2 *uvrA* did the two strains exhibit largely concordant responses (Table 2).

### 3.2. Comparisons of TA1535 versus TA100

Results similar to this comparison between TA1535 and TA100 were obtained in a previous evaluation, where 3.5% (23/659) of the compounds were considered mutagenic in TA1535 and clearly not mutagenic in TA100; the authors found that only 2.7% (18/659) were mutagenic in TA1535 and not in any other Ames strains [13]. In most of these cases, the compounds induced similar absolute increases in rev/plate in both strains, but the relative increases compared to the background were viewed by the authors as positive only in TA1535. However, acetaldehyde oxime, 6-mercaptopurine, and 1,3-butadiene produced higher absolute increases in rev/plate in TA1535 than in TA100. Methapyrilene has also been reported to be mutagenic in TA1535 but not in TA100 [14,15].

Only 36 of the 43 compounds that were mutagenic in TA1535 and not in TA100 in our dataset had data in other strains: 11 were mutagenic (albeit 2 in TA1537 only), and 26 were not in other strains. Thus, of the 1167 chemicals positive in TA100 or TA1535, only 26 (2%) were mutagenic only in TA1535 and not in any other strains based on the authors’ calls, virtually identical to the finding of Prival and Zeiger [13]. Both our analysis and that of Prival and Zeiger [13] document the generally greater sensitivity of TA100 relative to TA1535 and confirm that TA1535 provides little additional detection of bacterial mutagens to a test battery that already contains TA100, noted previously in another analysis [16].

### 3.3. Specificity of TA1535 for specific chemical structures using the Leadscope database

Although TA1535 is generally less sensitive than TA100, we wondered if it might show increased specificity for one or more chemical classes. Such a finding would make TA1535 critical for the detection of the mutagenicity of chemicals within those chemical classes—even though the analyses above show that only ∼2% of compounds are detected uniquely by TA1535 and not by any of the other OECD-recommended strains. We addressed this question by analyzing visually the chemical structures of the 43 mutagens that were positive in TA1535 but negative in TA100. The results showed that the chemicals occurred among seven chemical classes (Table 3).

More analyses are required to determine whether the presence of common classes amongst the chemicals positive in TA1535 but negative in TA100 indicates that the former strain has a unique ability to detect these classes; thus, we determined the percentage of mutagens in the Leadscope database for each class. The results show, for example, that the 3 oximes that are uniquely positive in TA1535 and not in TA100 account for 42.9% (3/7) of the mutagenic oximes in the Leadscope database (Table 3). Similarly, the 10 *N*- or *O*-allyl compounds or 3 base analogues that were uniquely positive in TA1535 and not in TA100 accounted for 37.5% and 33.3%, respectively, of compounds in these classes in the Leadscope database (Table 3). This implies that more than one-third of the mutagens within these chemical classes would be missed if TA1535 were not part of the test battery. In contrast, TA1535 had no specificity for nitro-compounds or nitrates and little for hydrazines (Table 3). Data could not be obtained for the phenobarbital class (consists only of two compounds: phenobarbital and primidone) or alkylating agents (chemicals too diverse to be amenable to a database search). However, in the latter case, it should be noted that there are several hundred mutagenic alkylating agents, thus the percentage truly selective in TA1535 is likely to be low.

For the remaining class (base analogues) few have been evaluated in the Ames strains for mutagenicity, and at least two that have been studied in detail, 6-hydroxylaminopurine and 2-amino-6-hydroxylaminopurine, are mutagenic in TA100 only because of the chance deletion of the *moeA* and *moaA* genes by the Δ(*gal bio uvrB*) mutation in that strain [17]. In addition, the chemical classes identified here are based on a retrospective analysis. Thus, TA1535 might have specificity for chemical classes not represented in our historic analysis, *e.g.*, proprietary pharmaceutical compounds not in our database.

### 3.4. Comparisons of TA1535 versus TA100 based on statistical analyses

The analyses described above relied on visual examination of the data and a qualitative determination of mutagenicity. However, OECD TG471 also allows for statistical analyses of dose-response data, which provide additional insight into the mutagenicity of agents. Statistical analyses can provide justification for pooling data from multiple experiments. In addition, the mutagenic potencies determined by such analyses permit one to make an objective and quantitative statement about the mutagenicity of an agent relative to other agents. Finally, a statistical analysis can help in the overall evaluation of the mutagenicity of an agent by indicating whether the mutagenicity of an agent is weak or strong relative to that of other agents.

Of the 43 compounds that we determined were mutagenic in TA1535 and not in TA100 based on the authors’ calls (Table 2), only 27 had dose-response data readily available. This included representatives from all the chemical classes listed in Table 3, although the coverage for *O*- and *N*-allyl compounds was low (2/10 compounds had dose-response data). Using those data to determine significant slopes (mutagenic potencies) from the linear regressions, one compound, *N*-butyl-2-nitroatoethylnitramine, was not mutagenic even in TA1535, and another, cyclohexanone oxime, was more mutagenic in TA100 than in TA1535 (Table 4). Thus, 63% (17/27) of the compounds were not selectively mutagenic in TA1535 relative to TA100 by this analysis. In fact, most of these compounds had similar mutagenic potencies in both TA1535 and TA100.

Although 37% (10/27) were mutagenic in TA1535 and not in TA100 by this analysis, the mutagenic potencies of 9 of these 10 compounds were extremely low, ranging from 0.001 to 0.05 rev/μg (Table 4). The only compound that was unambiguously mutagenic in TA1535 and not in TA100 was 6-mercaptopurine, which induced 7.5 (+S9) and 2.7 (−S9) rev/μg (Table 4). Based on this statistical analysis, removal of the 17 compounds from the 43 that we identified initially as selectively mutagenic in TA1535 and not in TA100 based on non-statistical evaluations showed that only 43 − 17 = 26 or 2.2% (26/1167) of the compounds for which there were data in both strains in the electronic databases were mutagenic in TA1535 and not in TA100.

We also examined the wider dataset of *in vitro* genotoxicity data for the 10 compounds that were mutagenic in TA1535 but not TA100 after statistical analysis. All the compounds had mutagenicity data from other Ames strains, and 2/10 were mutagenic in TA102 (hydrazine and acetaldehyde oxime). Most compounds (7/10) did not have data from either TA102 or *E. coli*. We also examined data for these compounds from other commonly used *in vitro* genotoxicity assays, including the micronucleus, chromosomal aberration, and mouse lymphoma assays. Adequate data in at least one of these assays were available for 6 compounds, 3 of which were mutagenic in one assay or another (hydrazine, phenobarbital, 6-mercaptopurine), and all three were mutagenic in the *in vitro* chromosomal aberration assay; 6-mercaptopurine was also mutagenic in the micronucleus and mouse lymphoma assays. In summation, 10 compounds were mutagenic in TA1535 but not TA100, 6 of these had adequate data from additional Ames strains or *in vitro* genotoxicity tests, and the activity of 4 out of those 6 compounds would have been detected through this wider evaluation.

An additional set of dose-response data for 53, anonymized, proprietary pharmaceuticals were contributed to determine if the trends observed above would also pertain to these compounds. There were data in both TA1535 and TA100 for only 43 of the 53 compounds, and statistical analyses indicated that 27.9% (12/43) were mutagenic in TA1535 and not in TA100 (Table 5). However, all 12 compounds had extremely low or marginal mutagenic potencies (≤0.05 rev/μg), with most having potencies of 0.002–0.009 rev/μg, as did those in our electronic databases. Thus, these 12 compounds exhibited potencies no different than those in our much larger electronic database.

Using the database from Gold and Zeiger [18], E. Zeiger (unpublished observations) found that the mutagenic potencies (rev/μg) in *Salmonella* of the mutagenic rodent carcinogens ranged from 0.003 to 3060, and those of the mutagenic non-carcinogens ranged from 0.001 to 42.5. Also, 21.4% (24/112) of the carcinogens had potencies ≤0.1 rev/μg, and 1 was > 1000. There were 22 mutagenic noncarcinogens, and 40.9% (9/22) had mutagenic potencies ≤0.1 rev/μg. In addition, 42.9% (48/112) and 31.8% (7/22) of the carcinogens and noncarcinogens, respectively, had mutagenic potencies > 0.7 rev/μg.

Thus, the low (< 0.05 rev/μg) mutagenic potencies exhibited by nearly all the compounds putatively viewed as mutagenic in TA1535 but not in TA100 places them at the bottom of the range of mutagenic potencies among a wide variety of agents evaluated for rodent carcinogenicity. Although there is no established correlation between mutagenic potency in *Salmonella* and carcinogenic potency in rodents, these data show that, except for 6-mercaptopurine, the agents putatively identified as mutagenic in TA1535 and not TA100 are extremely weak mutagens.

The mutagenic potencies (slope) of the linear regressions are the same whether the spontaneous number of rev/plate are subtracted from the zero and treatment doses prior to calculating the regression or whether the spontaneous value is retained at the zero and treatment doses. Thus, differing spontaneous values of the different strains do not influence the resulting mutagenic potencies. Indeed, as can be seen in Tables 4 and 5, the mutagenic potency of an agent is the same or similar in both TA1535 and TA100. This is partially because the compounds in these tables were a select group of agents that were perceived to be selectively mutagenic in TA1535. As our analysis showed, they were generally no more mutagenic in TA1535 than they were in TA100 and, with few exceptions, were not selectively mutagenic in TA1535.

### 3.5. Comparison of TA1537 versus TA97

TA97a is a reconstruction of TA97 from the Ames lab (B.N. Ames, personal communication), and the two strains are phenotypically identical. Thus, it was not always clear which strain a laboratory was using. Our initial query of the electronic databases found only 12 compounds tested specifically in both TA97 and TA97a. Thus, we pooled data for the two strains for the analysis here and refer only to TA97. After applying our selection criteria to the data in the Lhasa and Leadscope databases, we identified only 4% (10/247) of the compounds as mutagenic in TA1537 and not in TA97—regardless of any activity they may have in other strains (Table 2). There were no common chemical structures or chemical classes found by visual analysis of the structures for those 10 compounds, showing that TA1537 was not selective for a specific chemical class. Further analysis found that six of the compounds were mutagenic in other strains, whereas four were not. Thus, only 1.6% (4/247) of the compounds were mutagenic only in TA1537 and not in any other strains.

We found dose-response data for 9 of the 10 compounds reported by the authors to be mutagenic in TA15357 and not in TA97. Using those data to determine significant slopes (mutagenic potencies) from the linear regressions, we found that three compounds were mutagenic in TA1537 and not in TA97, four had similar mutagenic potencies in both strains, and two were not mutagenic in either strain (Table 6). The mutagenic potencies of the three TA1537-specific compounds were low to modest (0.005–0.2 rev/μg).

Chlorambucil is a well-known, highly potent germ-cell mutagen [19] that is clastogenic in a variety of genotoxicity assays [20] and was reported mutagenic in other Ames strains (*e.g.*, TA100 and TA102). Adequate data from other *in vitro* genotoxicity assays were not available for the remaining two TA1537-specific compounds (C.I. Basic Red 29 and Ro 60–0441). Additional Ames strain data were available for C.I. Basic Red 29, which produced equivocal results in TA100 and TA1535, and Ro 60–0441, which was not mutagenic in other strains, including TA98, TA100 and TA102.

In the initial paper that introduced strain TA97 [21], the authors evaluated 17 compounds and found none that were mutagenic only in TA1537 and not in TA97; instead, the compounds were generally more mutagenic in TA97 than in TA1537. Another study used statistical analyses to compare 13 compounds that were mutagenic in either TA97a and/or TA1537 and found that 23% (3/13) were mutagenic in only TA1537, and 15% (2/13) were mutagenic in only TA97a [22].

Because most study protocols do not include tests in both TA97 and TA1537, data used to compare responses in these strains were often taken from studies conducted in different laboratories, which could be a confounding factor in our analysis. Thus, we subjected for additional testing five chemicals identified initially as mutagenic selectively in TA1537. In all five cases, assays conducted with these high-purity chemicals under identical conditions in a modern laboratory produced concordant results, *i.e.* chemicals were either mutagenic in both TA97 and TA1537 (altertoxin I, C.I. Basic Red 29) or not mutagenic in either TA97 or TA1537 (chlorambucil, 2-methylpropanenitrile, retrorsine) (Table 6). There were no selective mutagenic responses in TA1537 even when using the less stringent 2- or 3-fold rule, which included 2 of the 3 compounds confirmed by statistical analysis to be selective TA1537 mutagens based on published data [23].

In most of the pairwise analyses reported here, quantitative analysis of the dose-response data using statistical techniques showed that reported selective responses in test strains were usually weak and/or replicated in the comparator strain. It is possible that this finding is an artefact of the analysis and that similar results may be obtained from an analysis of selective responses in comparator strains. To evaluate this, we analyzed a dataset of 16 compounds that were reported to be mutagenic in TA97 and for which there were dose-response data both in TA97 and TA1537. Our analysis showed that one of the compounds was not mutagenic in either strain, two were more potent in TA97, and the remaining 13 were mutagenic in TA97 and not in TA1537 (Table 7).

### 3.6. Comparison of E. coli WP2 strains versus Salmonella TA102

Using the calls reported by the authors in studies available from the two electronic databases, we found only a few compounds for which there were data in the *E. coli* WP2 strains and/or TA102 present in the same study. For the various pairwise comparisons, we found (1) no compounds that were mutagenic in *E. coli* WP2 *uvrA* but not in *E. coli* WP2 *uvrA* pKM101, (2) 4/46 that were mutagenic in TA102 but not in *E. coli* WP2 *uvrA* pKM101, and (3) similar responses for *E. coli* WP2 *uvrA* and TA102, with only three compounds selectively mutagenic in the former strain and four in the latter (compared to 27 compounds mutagenic in both). Overall, the data indicated that *E. coli* WP2 *uvrA* pKM101 had higher sensitivity than the other two strains. Thus, we pooled the data from TA102 and *E. coli* WP2 *uvrA* (pooled test strain) and compared the responses to those in *E. coli* WP2 *uvrA* pKM101 (comparator strain). There were only four compounds that were mutagenic in either or both pooled test strains and not in the comparator strain: danthron, emodin, mitomycin-C, and bleomycin.

Statistical analysis confirmed the selective responses in the pooled strains, with mutagenic potencies (rev/μg) ranging from 6.6 ± 2.0 for emodin to 14,036 ± 1196 for mitomycin C. Both danthron and emodin are anthraquinones and are mutagenic in other Ames strains, indicating that neither TA102 nor *E. coli* WP2 *uvrA* exhibit any selectivity for detecting anthraquinones. Both anthraquinones, mitomycin-C and bleomycin, are clastogens detected by the chromosomal aberration test and/or micronucleus assay. In summary, danthron and emodin are detected as mutagenic by other bacterial strains, and mitomycin-C and bleomycin are detected by another assay recommended in commonly used genotoxicity test batteries such as that recommended in ICH S2(R1) [24]. Bleomycin and mitomycin C are potent cross-linking agents that require a *uvr+* genetic background for resolution of crosslinks that otherwise inhibit cell division and mutant expression.

Given the small size of the data set under comparison, we decided to augment the analysis by generating data for five additional compounds with incomplete or inconsistent data in TA102, *E. coli* WP2 *uvrA* pKM101 and *E. coli* WP2 *uvrA* [23]. The findings from the analysis of public data were supported by our newly generated data for 4/5 of those compounds (Table 8). Two produced concordant positive results in all strains (folpet and diethyl nitrosamine), and one produced concordant negative results (NiCl_2_). Additionally, one compound (*p*-toluenesulfonyl hydrazide) was negative in TA102 but positive in both strains of *E. coli.* Only one compound failed to follow this trend (acrylonitrile), which was positive in TA102 and *E. coli* WP2 *uvrA* but not *E. coli* WP2 *uvrA* pKM101 and is also reported to be positive in TA100. Hence, even with a larger dataset, there were no compounds that would not be detected by *E. coli* WP2 *uvrA* pKM101, other Ames strains, or additional genotoxicity assays, supporting the redundancy of TA102 and *E. coli* WP2 *uvrA*.

There were four published studies that compared results in the three strains [25–28], and these are summarized in Table 9. Overall, these analyses indicated that *E. coli* WP2 *uvrA* pKM101 detected more mutagens selectively, (7 + 4)/52 = 21.2%, than TA102, (2 + 3)/ 52 = 9.6%. Statistical analysis of the data for the seven compounds that were mutagenic in WP2 *uvrA* pKM101 but not in TA102 in Watanabe et al. [25] showed that 6/7 had mutagenic potencies ≤0.05 rev/μg; the seventh, isonicotinaldoxime, induced 0.27 rev/μg in WP2 *uvrA* pKM101. Statistical analysis of the data for the four compounds that were mutagenic in WP2 *uvrA* pKM101 but not in TA102 in Watanabe et al. [26] showed that one (allyl chloride) was not mutagenic in WP2 *uvrA* pKM101. The remaining three compounds were mutagenic in WP2 *uvrA* pKM101, with potencies ranging from 0.5 to 5 rev/μg.

### 3.7. Assessment of sensitivity when testing using only strains TA98 and TA100

**Fig. 3.**
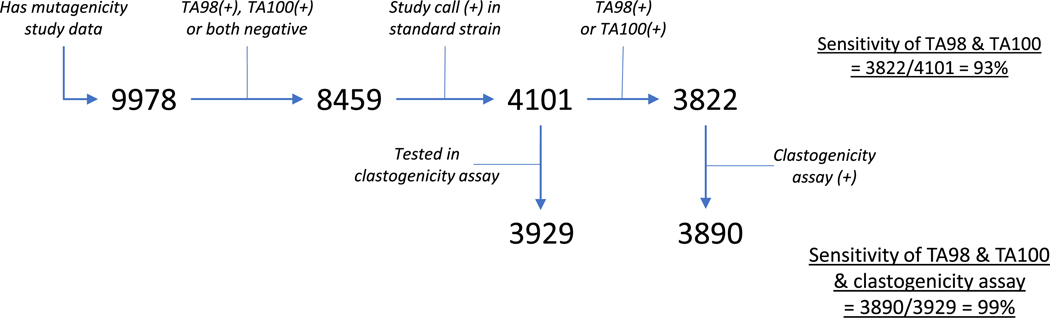
Schematic diagram showing 99% sensitivity when testing using only TA98 and TA100 and including the clastogenicity assays *versus* the standard battery of bacterial strains. Additional criteria are iteratively applied to the same compound dataset.

To assess the sensitivity of bacterial mutagenicity testing using only strains TA98 and TA100, we performed the following analysis, which is outlined in Fig. 3. Out of 9978 compounds in the Leadscope database with bacterial mutation study calls, 8459 were mutagenic in TA98 and/or TA100, or they were not mutagenic in either strain. Of these, 4135 compounds were classified as mutagenic studies due to positive results in at least one bacterial strain, and 3822 were mutagenic in either TA98 or TA100.

Of the 313 (4135 − 3822) mutagens that did not generate a positive result in either TA98 or TA100, 34 were mutagenic only in bacterial strains other than those recommended by TG471 (primarily TA104), whereas the remaining 279 chemicals were found to be mutagenic in other TG471 strains. Thus, there were 4101 (4135 − 34) compounds that were either mutagenic in strains TA98 and/or TA100, or not mutagenic in either strain but mutagenic when tested in other TG471-recommended bacterial strains. This calculation yielded a sensitivity when testing with only TA98/TA100 of 93% (3822/4101). Thus, using only TA98 and TA100 would permit the detection of 93% of the bacterial mutagens detected collectively by all the TG471-recommended bacterial strains.

Finally, we determined how many of the bacterial mutagens missed by using only TA98 and TA100 would be detected if testing included either the mouse lymphoma assay (MLA) or *in vitro* chromosomal aberration (CA) tests in mammalian cells, both of which can detect clastogens. Among the 4101 compounds with data in TA98 or TA100 curated as described above, our database had MLA or CA data on all but 172, leaving 4101 − 172 = 3929 bacterial mutagens with curated data in TA98 or TA100 that also had MLA or CA data; this number formed the denominator for assessment. We identified 68 additional compounds that were not mutagenic in either TA98 or TA100 but displayed activity in at least one of the *in vitro* clastogenesis assays; therefore, 3822 + 68 = 3890 compounds formed the numerator. Consequently, 99% (3890/3929) of bacterial mutagens detected by using all the TG471-recommended bacterial strains would be detected by using just strains TA98 and TA100 plus one of the recommended *in vitro* clastogenesis assays.

In this analysis we did not restrict our data to only studies conducted in all 4 or 5 standard TG471 strains. We subsequently evaluated the performance of using TA98/TA100 with just the subset of NTP study data of 1505 compounds where all 5 strains were tested. This dataset has the advantage of being better curated and peer reviewed. However, it is a very small part of the entire set, is skewed towards non-mutagenic compounds, and represents a less diverse chemical space compared to the entire set. In this subset, 84% (198/236) of the bacterial mutagens were detected by TA98 or TA100 or both. This result revealed that as more testing has been performed over time, leading to a greater than 6-fold increase in the number of chemicals analyzed, sensitivity using just TA98/TA100 strain testing has increased over that calculated earlier. Additionally, the current, larger dataset reflects recent chemistry and is more relevant to continued assay application.

## 4. Conclusions

Three questions were examined initially, which we repeat here for clarity: (1) Although TA100 is derived from TA1535 and generally detects more mutagens than TA1535, does TA1535 detect any mutagens that are not detected by TA100? (2) Because there is a choice to use one of either TA1537, TA97 or TA97a, are these strains truly equivalent? (3) Because there is a choice to use one of either *E. coli* WP2 *uvrA*, *E. coli* WP2 *uvrA* pKM101, or TA102, are these strains truly equivalent? Table 10 details most of the steps involved in the analysis of these questions.

Firstly, there were few mutagens that displayed selective activity in our test strains (43 in TA1535, 10 in TA1537, and 4 in a combination of *E. coli* WP2 *uvrA* and TA102) and not in our comparator strains (TA100, TA97 and *E. coli* WP2 *uvrA* pKM101 respectively). Secondly, there were even fewer mutagens that displayed both selective and unique activity (26, 5, and 2, respectively). Thirdly, when a statistical test was used to determine whether responses in test and comparator strains were quantitatively similar, the number of selective and unique chemicals fell further (8, 2, and 2, respectively). Lastly, if we considered responses in other commonly used *in vitro* genotoxicity assays, then the number of mutagens that would fail to be detected by the removal of TA1535, TA1537, *E. coli* WP2 *uvrA*, and TA102 dropped to only 2 in total, from the > 1000 mutagens with which we commenced this analysis. Thus, the answers to our questions, based on this analysis of publicly available data are (1) using TA100 alone would suffice, (2) TA97 should be used in preference to TA1537, and (3) *E. coli* WP2 *uvrA* pKM101 should be used in preference to *E. coli* WP2 *uvrA* and TA102.

These outcomes are supported by further work: an analysis of proprietary data for 53 compounds tested in both TA100 and TA1535, which produced concordant results with our analysis of publicly available data. Additional tests conducted on five compounds that were purportedly mutagenic in TA1537 and not in TA97 based on historical data were shown to produce concordant results in both TA1537 and TA97, as were five additional compounds with incomplete or inconclusive data in *E. coli* WP2 *uvrA*, *E. coli* WP2 *uvrA* pKM101, and TA102. In the latter case, the activity of 4/5 compounds was detected by *E. coli* WP2 *uvrA* pKM101, with the one compound also not detected by TA102.

Finally, an analysis of the performance of strains TA98 and TA100 alone indicated that these were enough for detecting most bacterial mutagens (93%); agreeing with conclusions of a previous analysis [16]. When including an *in vitro* assay that detects clastogens such as the *in vitro* chromosome aberration assay [24], the resultant battery would detect 99% of bacterial mutagens, *i.e.*, those identified as such by using all the bacterial strains recommended by TG471.

Overall, these analyses support a conclusion that *Salmonella* strains TA1535, TA1537, TA102, and *E. coli* strain WP2 *uvrA* could be removed from those recommended in OECD TG471 with little, if any, loss of sensitivity for the detection of bacterial mutagens. Modification of testing based upon these findings could reduce the cost, time, and redundancy of the current OECD Test Guideline TG471.

## Figures and Tables

**Table 1:** Numbers of compounds in the Lhasa and Leadscope datasets for each comparison.

		Number of compounds	
Test strain	Comparator strain	Lhasa	Leadscope

TA1535	TA100	3667	4701
TA1537	TA97/TA97a	633	993
TA102	WP2 *uvrA* pKM101	148	150
TA102	WP2 *uvrA*	135	318
WP2 *uvrA*	WP2 *uvrA* pKM101	129	102

**Table 2:** Selectivity of test strains after merging Leadscope and Lhasa datasets and imposing selection criteria.

		No. of compounds whose selectivity was re-evaluated				No. of compounds	
Strain comparison (test-comparator)	Total no. of compounds^a^	Not mutagenic^b^	Inconsistent responses^c^	Dissimilar protocol^d^	Not selective^e^	confirmed as selective in test strain	Selectivity^f^ (%)

TA1535-TA100^g^	1231	28	27	2	37	43	43/1167 (3.7)
TA1537-TA97	271	2	15	7	7	10	10/247 (4.0)
WP2-WP2p^h^	25	0	3	1	0	0	0/21 (0.0)
TA102-WP2p	49	1	2	0	0	4	4/46 (8.0)
TA102-WP2	43	2	4	3	0	3	3/34 (8.8)

a Number of compounds reported to produce a positive result in either test or comparator strain.

b Not positive in test strain.

c Inconsistent responses in test or comparator strains that could not be explained by the protocol.

d Dissimilar protocols, which prevented comparison.

e Not selective, *i.e.*, the compound was mutagenic in both strains.

f Denominator = total number of compounds less those not mutagenic, producing inconsistent responses or from dissimilar protocols.

g We could not classify 7 compounds in this comparison due to lack of papers or data; these and one duplicate compound were also removed from the denominator.

h WP2p, *E. coli* WP2 *uvrA* pKM101; WP2, *E. coli* WP2 *uvrA*.

**Table 3:** Chemical classes of the 43 compounds confirmed from the electronic dataset as mutagenic in TA1535 and not in TA100.

Chemical class or model compound	Number of compounds	% of mutagens in each chemical class with TA1535 uniquely positive compounds (Leadscope database^a^)

*N*- or *O*-Allyl	10/27	37.5
Alkylating agent	8	Not applicable
Base analogue	3/9	33.3
Oxime	3/7	42.9
Nitro or nitrate	3/238	1.26
Hydrazine	3/20	15.0
Phenobarbital	2	Not applicable
Miscellaneous	11	Not applicable

a For example, the 3 oximes mutagenic in TA1535 and not in TA100 account for 42.9% of the oximes in the Leadscope database tested in both TA1535 and TA100.

**Table 4:** Mutagenic potencies (rev/μg) of compounds reported by the authors as mutagenic in TA1535 and not in TA100.

	TA1535		TA100		
Compound	+S9	-S9	+S9	-S9	Outcome

2-(Methylnitroamino)ethyl nitrate	0.001	0.009	-	-	TA1535 Selective
4-Ethylmorpholine	0.02	-	-	-	TA1535 Selective
6-Mercaptopurine	7.5	2.7	-	-	TA1535 Selective
Acetaldehyde oxime	ND^a^	0.02	ND^a^	-	TA1535 Selective
Carbonochloridothiolic acid	0.05	ND^a^	-	-	TA1535 Selective
Dichloro(methyl)(vinyl) silane	0.05	ND^a^	-	ND^a^	TA1535 Selective
Hydrazine sulfate	ND^a^	0.05	ND^a^	-	TA1535 Selective
Phenobarbital	ND^a^	0.02	-	-	TA1535 Selective
Tricaprylin	0.004	0.004	-	-	TA1535 Selective
Acetin	0.01	0.01	-	-	TA1535 Selective
1H-Benzotriazole	0.02	0.01	0.02	0.02	Similar response
Bromochlorodifluoromethane	3.8	1.8	-	1.3	Similar response
1,2-Dibromopropane	0.02	0.02	0.2	-	Similar response
2-Bromopropionic acid	0.01	ND^a^	0.02	ND^a^	Similar response
1-Bromotetrafluoro-2-iodoethane^b^	6950^b^	7400^b^	-	5500^b^	Similar response
Dexlansoprazole	ND^a^	0.05	-	0.07	Similar response
Maltol	0.01	-	0.02	0.02	Similar response
Methyl ethyl ketoxime	0.01	-	0.01	ND^a^	Similar response
Oxetane	0.006	-	-	0.002	Similar response
Pentaerythritol triallyl ether	ND^a^	0.01	ND^a^	0.01	Similar response
Primadone	ND	0.003	ND^a^	0.003	Similar response
Roche 47 3555	0.01	0.002	0.02	-	Similar response
Sec-Butyl nitrite	0.01	-	0.01	0.03	Similar response
Thiourea dioxide	0.02	0.02	0.01	-	Similar response
Trimethylolpropane triacrylate	0.01	-	0.01	-	Similar response
*N*-Butyl-2-nitratoethylnitramine	-	-	-	-	Not mutagenic
Cyclohexanone oxime	0.02	-	-	0.5	TA100 greater

a ND = no data.

b Rev/% concentration.

**Table 5:** Mutagenic potencies (rev/μg) of anonymized, proprietary pharmaceuticals.

	TA1535		TA100		+ in only
Chemical	-S9	+S9	-S9	+S9	TA1535

1	-	-	-	-	
2	-	0.12	ND	ND	
3	-	0.005	-	-	X
4	0.02	0.01	-	0.01	
5	-	0.02	ND	ND	
6	-	-	0.007	-	
7	0.003	0.009	-	-	X
8	0.004	-	-	-	X
9	0.01	0.01	0.01	0.01	
10	0.02	0.01	0.02	0.01	
11	-	ND^a^	0.003	ND	
12	-	0.002	-	-	X
13	0.01	0.01	0.01	0.01	
14	-	0.005	-	0.004	
15	-	-	ND	ND	
16	-	-	-	-	
17	-	-	-	-	
18	-	-	-	-	
19	-	-	-	-	
20	0.01	0.02	-	0.86	
21	0.01	0.01	ND	ND	
22	0.002	0.002	-	-	
23	0.0007	0.03	-	0.01	
24	0.001	0.01	-	0.01	
25	0.01	0.007	-	-	X
26	0.01	0.01	-	-	X
27	0.01	0.01	ND	ND	
28	0.05	0.05	0.02	0.01	
29	0.05	0.05	0.01	0.01	
30	0.06	0.06	-	0.06	
31	0.01	0.04	ND	ND	
32	-	0.002	-	-	X
33	-	-	ND	ND	
34	-	-	-	-	
35	-	-	-	-	
36	0.002	0.002	-	0.01	
37	0.004	0.004	-	-	X
38	0.003	-	-	-	X
39	0.003	-	ND	ND	
40	0.007	ND	0.05	ND	
41	-	0.15	-	-	X
42	0.006	0.006	0.006	0.02	
43	-	0.05	-	-	X
44	-	0.005	-	0.006	
45	-	0.005	ND	ND	
46	0.01	0.02	0.02	0.02	
47	-	-	-	-	
48	0.03	-	-	-	X
49	0.002	0.01	-	0.003	
50	-	0.004	-	0.03	
51	ND	0.01	ND	ND	
52	-	-	-	-	
53	0.04	ND	ND	ND	

a ND = No data.

**Table 6:** Mutagenic potencies (rev/μg) in TA1537 and TA97 of compounds identified in the electronic dataset as mutagenic only in TA1537, and qualitative results from concurrent re-evaluation.

	Evaluation of compounds in electronic dataset					Re-evaluation^a^			
	TA1537^a^		TA97^a^			TA1537		TA97	
Compound	+S9	-S9	+S9	-S9	Outcome	+S9	-S9	+S9	-S9

C.I. Basic Red	0.2	0.2	–	–	TA1537 selective	+	+	+	+
Chlorambucil	0.005	ND^b^	–	ND	TA1537 selective	–	–	–	–
Ro 60–0441	0.1	0.05	–	–	TA1537 selective	ND	ND	ND	ND
Altertoxin I	1.7	3.2	–	1.3	Similar responses	+	+	+	+
Amiloride hydrochloride	ND	0.1	ND	0.09	Similar responses	ND	ND	ND	ND
1-(4-Bromophenyl)-3,3-triazine	1.5	0.05	1	1	Similar responses	ND	ND	ND	ND
2-Methylpropanenitrile	–	0.04	–	0.009	Similar responses	–	–	–	–
Z-2,3-Dimethyloxypropenylbenzene	ND	–	ND	–	Not mutagenic	ND	ND	ND	ND
Retrorsine	–	ND	–	ND	Not mutagenic	–	–	–	–

a Data from [23].

b No data.

**Table 7:** Mutagenic potencies (rev/μg) in TA1537 and TA97 of compounds identified in published reports as mutagenic in TA97 where there were also dose-response data in TA1537.

	TA97		TA1537		
Compound	+S9	-S9	+S9	-S9	Outcome

Benzo(f)-quinoline	6	0	-	-	TA97 selective
1,2-Dibromo-3-chloropropane	22	-	-	ND^a^	TA97 selective
Dimethylolurea	0.1	-	-	-	TA97 selective
Ethylene glycol diethyl ether	-	0.01ND^a^		-	TA97 selective
Formaldehyde	3.3	-	-	-	TA97 selective
4,4’-Methylenebis(2-chloroanaline)	1	-	-	ND^a^	TA97 selective
α-Naphthyl isothiocyanate	2	-	-	ND^a^	TA97 selective
Piperonol	0.3	0.2	-	-	TA97 selective
Potassium bromate	0.1	0.04-		-	TA97 selective
Propylene glycol mono-t-butyl	-	0.02ND^a^		-	TA97 selective
3,3’,4,4’-Tetrachloroazobenzene	2	-	-	ND^a^	TA97 selective
4,4’-Thiodianiline	100	-	-	-	TA97 selective
3,4-Xylidine	0.4	-	-	ND^a^	TA97 selective
Nitrofurantoin	432	1500	1.2	-	TA97 stronger
1,2,3-Trichloropropane	3.9	-	0.03	-	TA97 stronger
2,4-Xylidine	-	-	-	ND^a^	Not mutagenic

a No data.

**Table 8:** Qualitative results for compounds re-evaluated concurrently in TA102-WP2 strains.^a^

	TA102		WP2 *uvrA* pKM101		WP2 *uvrA*	
Compound	−S9	+S9	−S9	+S9	−S9	+S9

Acrylonitrile	+	+	-	-	+	+
*N,N’*-Diethylnitrosamine	NP^b^	+	NP	+	NP	+
Folpet	+	+	+	+	+	+
NiCl_2_	-	-	-	-	-	-
*p*-Toluenesulfonyl hydrazide	-	-	-	+	+	+

a Data from [23].

b Re-evaluation experiments not performed.

**Table 9:** Number of compounds evaluated in both TA102 and WP2 uvrA pKM101 in the same studies.^a^

Classification group	Watanabe et al. [25]	Watanabe et al. [26]	Watanabe II et al. [27]	Wilcox et al. [28]

Mutagenic in both strains	10	14	6	6
Mutagenic only in TA102	0	2	0	3
Mutagenic only in WP2 *uvrA* pKM101	7	4	0	0

a Calls made by the authors; only Wilcox et al. [28] used a statistical analysis.

**Table 10:** Number of compounds mutagenic in specific strains/assays.

		Number of compounds			
Strain comparison (test-comparator)	Types of analyses	+ in test strain, - in comparator	+ in test strain, - in other strains	+ in test strain, - in other genetox assays	+ only in test strain

TA1535-TA100	Reviewed data	43	26^b^	4^c^	3
	After statistics	10	8	2	2
TA1537-TA97	Reviewed data	10	5	0^d^	0
	After statistics	3	2	0	0
WP2/TA102-WP2p^a^	Reviewed data	4	2	0	0
	After statistics	4	2	0	0

a Test strain = pooled data for *E. coli* WP2 *uvrA* and TA102, comparator strain = *E. coli* WP2 *uvrA* pKM101.

b Only 35 chemicals had data for additional Ames strains.

c Only 22 chemicals had adequate data in other genetox assays *in vitro*.

d Only 3 chemicals had adequate data in other genetox assays *in vitro*.
